# Safety of masitinib in patients with neurodegenerative diseases: a meta-analysis of randomized controlled trials

**DOI:** 10.1007/s10072-024-07502-y

**Published:** 2024-04-17

**Authors:** Abdullah Ashraf Hamad, Basma Ehab Amer

**Affiliations:** 1 Faculty of Medicine, Menoufia University, Menoufia, Egypt; 2 Faculty of Medicine, Benha University, Benha, Egypt; 3 Medical Research Group of Egypt, Negida Academy, Arlington, MA USA

**Keywords:** Masitinib, Neurodegeneration, Meta-analysis, Multiple sclerosis, Alzheimer's disease, ALS

## Abstract

**Objectives:**

This meta-analysis aimed to examine the safety of masitinib in patients with neurodegenerative diseases.

**Methods:**

We considered randomized controlled trials (RCTs) comparing different doses of masitinib versus placebo. We performed our analysis using the R (v.4.3.0) programming language and the incidence of adverse events was pooled using risk ratio (RR) and 95% confidence interval (CI).

**Results:**

We included five RCTs, focusing on multiple sclerosis (MS), Alzheimer's disease (AD), and amyotrophic lateral sclerosis. The meta-analysis revealed a significantly higher incidence of adverse events in the masitinib group compared to the control group, regardless of adverse event grade and masitinib dose (RR = 1.12, 95% CI [1.07 to 1.17], *P* < 0.01). Adverse events categorized as severe, non-fatal serious, leading to dose reduction, and leading to permanent discontinuation also showed a higher incidence in the masitinib group (*P* ≤ 0.01). Subgroup analysis for AD and MS supported these findings. The pooled incidence of adverse events, regardless of their grade, was higher in the masitinib group for both the 3 mg/kg/d dose (RR = 1.13, *P* = 0.01) and the 4.5 mg/kg/d dose (RR = 1.11, *P* < 0.01). However, there was no significant difference between masitinib 3 mg/kg/d dose and placebo regarding severe and non-fatal serious adverse events for the.

**Conclusion:**

Masitinib use in neurodegenerative diseases presents safety concerns that may impact patients' quality of life and require management. Further research is recommended to determine the optimal dose with minimal safety concerns in this patient population.

**Supplementary Information:**

The online version contains supplementary material available at 10.1007/s10072-024-07502-y.

## Introduction

Masitinib is a tyrosine kinase inhibitor initially developed as an anticancer drug [1, 2]. However, its potential neuroprotective effects prompted its investigation in neurodegenerative diseases [3]. In a recently published scoping review by Hamad et al., the neuroprotective activities of masitinib were demonstrated through preclinical and clinical studies, showing its impact on cell proliferation and survival, reduction of neuroinflammation, and antioxidant activity [3]. Despite the promising therapeutic benefits observed, several clinical studies have raised concerns about the safety of masitinib [3]. In this article, we aim to expand on the safety profile by conducting the first meta-analysis of masitinib in patients with neurodegenerative diseases.

## Methods

This meta-analysis builds upon a previous systematic scoping review of masitinib's neuroprotective activities in preclinical and clinical studies [3]. The original study provides detailed information on literature search, screening, and inclusion criteria. In summary, four databases were searched using masitinib-related terms from inception to August 2023. Two authors independently conducted screening to identify relevant clinical and preclinical studies. Sixteen studies were identified, including five randomized controlled trials (RCTs) that were included in this meta-analysis. Of these RCTs, two focused on multiple sclerosis (MS) [4, 5], two on Alzheimer's disease (AD) [6, 7], and one on amyotrophic lateral sclerosis [8]. Characteristics of the RCTs and the participants were also described in detail in the original study [3].

For the meta-analysis, adverse event data were independently extracted by the two authors using an online data extraction form. The data were categorized based on adverse event grade (any grade, severe, non-fatal serious, leading to dose reduction, leading to permanent discontinuation, and leading to death) and masitinib dose (3, 4.5, and 6 mg/kg/d). All analyses were performed using the R (v.4.3.0) programming language and the "meta" package of RStudio software for Windows. The random effect model was employed to account for any suspected heterogeneity, and the incidence of adverse events was pooled using risk ratio (RR) and 95% confidence interval (CI). Inconsistency across studies was assessed using the I-squared (I^2^) test. Subgroup analysis was conducted based on the specific neurodegenerative disease and masitinib's dose.

## Results

### Overall findings regardless of dose

**Fig. 1:**
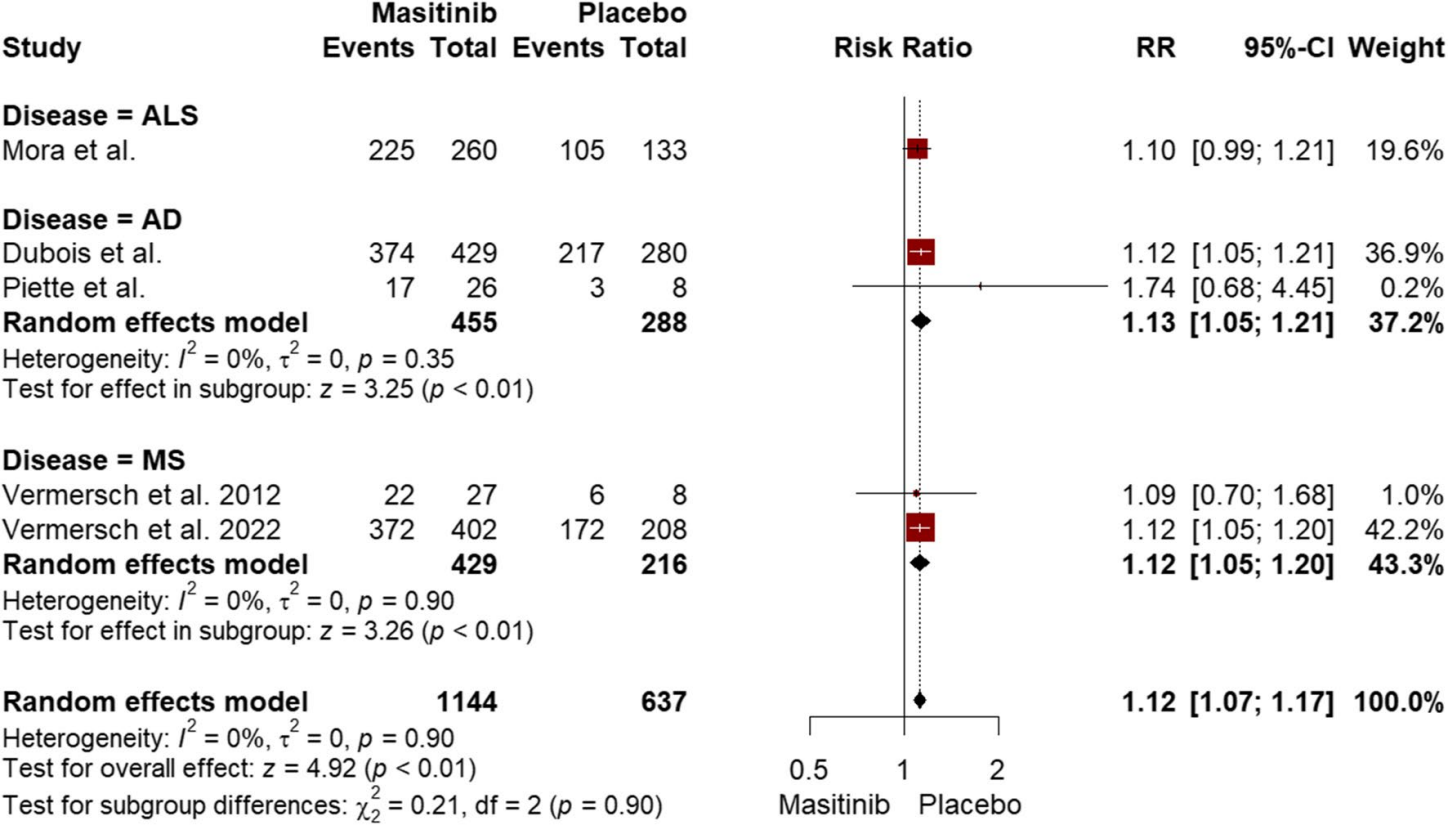
Forest plot comparing the overall incidence of adverse events, irrespective of their severity and masitinib dosage, between the masitinib and placebo groups

The pooled incidence of adverse events, regardless of their grade and masitinib dose, was significantly higher in the masitinib group (RR = 1.12, 95% CI [1.07 to 1.17], *P* < 0.01), with low heterogeneity observed (I^2^ = 0%, *P* = 0.90) (Fig. 1 & Table 1**)**. This significant difference was also observed in the subgroup analysis for AD and MS (RR = 1.13, 95% CI [1.05 to 1.21], P < 0.01; RR = 1.12, 95% CI [1.05, 1.20], P < 0.01, respectively), as shown in Fig. 1. Regarding severe adverse events, the pooled incidence was higher in the masitinib group (RR = 1.36, 95% CI [1.07 to 1.73], *P* = 0.01), with low heterogeneity between studies (I^2^ = 0%, *P* = 0.82) (Supplementary eFigure 1 & Table 1). The pooled incidence of non-fatal serious adverse events was higher in the masitinib group compared to the placebo, both overall and when subgrouped for AD and MS (*P* < 0.01) (Supplementary eFigure 2 & Table 1). Likewise, the incidence of adverse events leading to dose reduction or discontinuation was higher in the masitinib group (RR = 2.43 and 3.22, respectively, *P* < 0.01) (Supplementary eFigure 3 & Supplementary eFigure 4 & Table 1). However, there was no significant difference between the two groups regarding adverse events leading to death (RR = 0.87, 95% CI [0.47 to 1.61], *P* < 0.66) (Supplementary eFigure 5 & Table 1).

**Table 1:** Summary of our subgroup analysis based on different masitinib doses

Adverse effect	Dose (mg/kg/d)	Number of studies	Group size (masitinib/placebo)	Risk Ratio	95% Confidence Interval	*P *value	Heterogeneity P value
Any grade	Any dose	5	1144/637	1.12	[1.07 to 1.17]	< 0.01	0.90
	3	2	189/413	1.13	[1.03 to 1.24]	0.01	0.21
	4.5	3	513/514	1.11	[1.05 to 1.17]	< 0.01	0.81
Severe	Any dose	4	742/429	1.36	[1.07 to 1.73]	0.01	0.82
	3	2	189/413	1.08	[0.66 to 1.77]	0.75	0.22
	4.5	2	314/413	1.50	[1.14 to 1.98]	< 0.01	0.38
Non-fatal serious	Any dose	5	1144/637	1.80	[1.40 to 2.32]	< 0.01	0.71
	3	2	189/413	1.39	[0.91 to 2.13]	0.13	0.42
	4.5	3	513/514	1.84	[1.36 to 2.50]	< 0.01	0.60
Leading to dose reduction	Any dose	2	286/141	2.43	[1.43 to 4.11]	< 0.01	0.86
Leading to discontinuation	Any dose	4	1118/629	3.22	[2.05 to 5.07]	< 0.01	0.13
	3	2	189/413	2.76	[1.21 to 6.32]	0.02	0.05
	4.5	3	513/514	3.17	[1.55 to 6.48]	< 0.01	0.06
Leading to death	Any dose	3	1091/621	0.87	[0.47 to 1.61]	0.66	0.82
	3	2	189/413	0.96	[0.45 to 2.05]	0.92	0.72
	4.5	3	513/514	0.78	[0.35 to 1.71]	0.53	0.36

### Subgroup analysis by dose

**Fig. 2:**
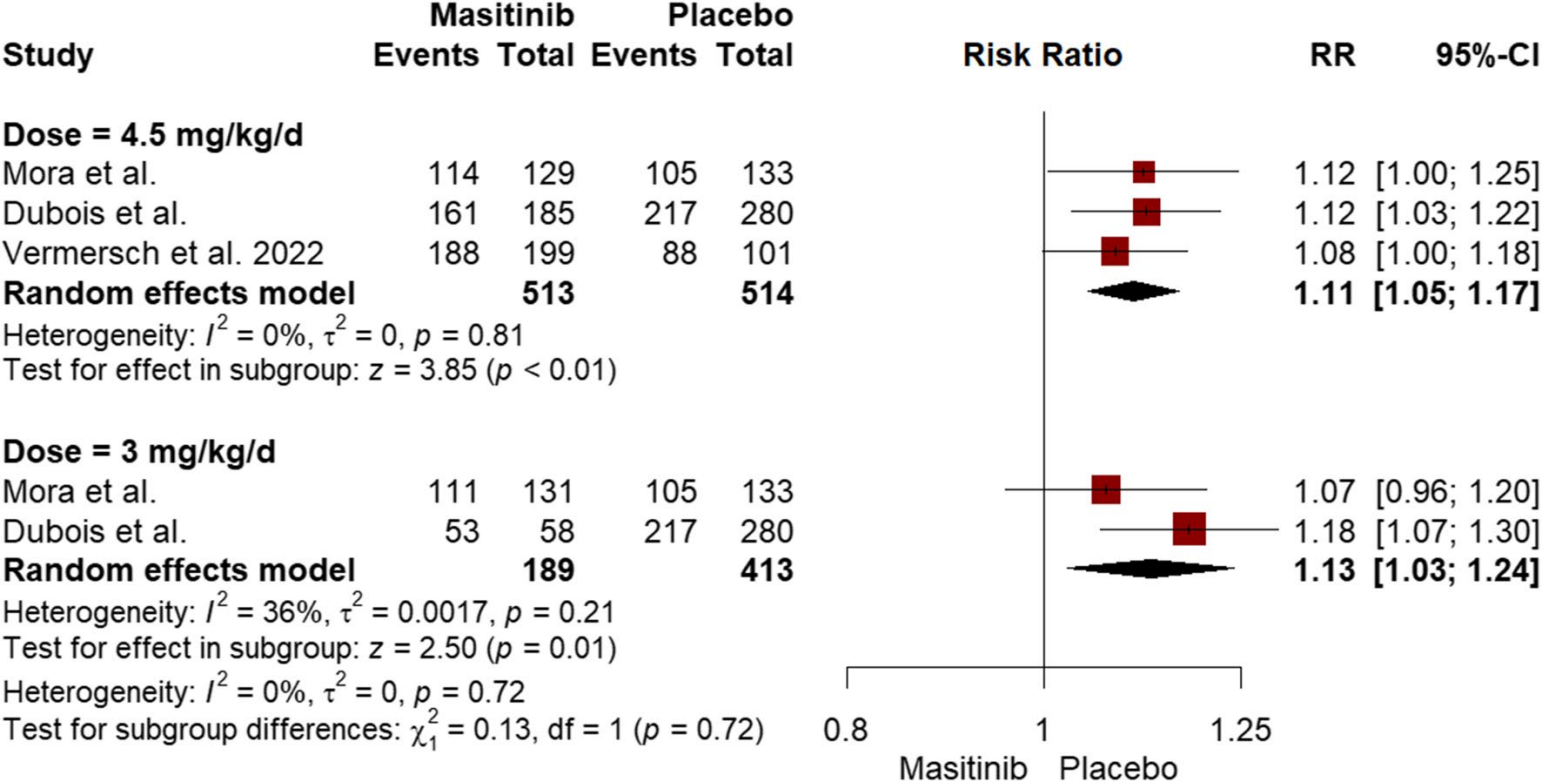
Forest plot of subgroup analysis for masitinib dosage, comparing the combined incidence of adverse events, irrespective of their severity, between the masitinib and placebo groups

Regardless of their grade, the pooled incidence of adverse events was higher in the masitinib group for both the 3 mg/kg/d dose (RR = 1.13, 95% CI [1.03 to 1.24], *P* = 0.01) and the 4.5 mg/kg/d dose (RR = 1.11, 95% CI [1.05 to 1.17], *P* < 0.01) (Fig. 2 & Table 1**)**. The incidence of severe adverse events did not differ significantly between masitinib 3 mg/kg/d dose and placebo (RR = 1.08, 95% CI [0.66 to 1.77], *P* = 0.75). However, the incidence of severe adverse events was higher with masitinib 4.5 mg/kg/d dose (RR = 1.50, 95% CI [1.14 to 1.98], *P* < 0.01) compared to placebo (Supplementary eFigure 6 & Table 1). Similarly, no significant difference was observed between masitinib 3 mg/kg/d dose and placebo regarding non-fatal serious adverse events (RR = 1.39, 95% CI [0.91 to 2.13], *P* = 0.13), but it was higher with the 4.5 mg/kg/d dose compared to placebo (RR = 1.84, 95% CI [1.36 to 2.50], *P* < 0.01) (Supplementary eFigure 7 & Table 1). Adverse events leading to treatment discontinuation were more frequent with both masitinib doses (3 and 4.5 mg/kg/d) compared to the placebo (RR = 2.76, 95% CI [1.21 to 6.32], *P* = 0.02; RR = 3.17, 95% CI [1.55 to 6.48], *P* < 0.01, respectively) (Supplementary eFigure 8 & Table 1). In contrast, both doses were comparable to placebo in terms of adverse events leading to death (Supplementary eFigure 9 & Table 1).

## Discussion

The present meta-analysis aimed to evaluate the safety profile of masitinib in patients with neurodegenerative diseases through a comprehensive analysis of RCTs. The findings of this meta-analysis demonstrate that masitinib is associated with a higher incidence of adverse events compared to placebo across different neurodegenerative diseases.

The overall pooled analysis revealed a significantly higher incidence of adverse events in the masitinib group compared to the placebo group, regardless of the dose administered. The incidence of all grades of adverse events was significantly higher in the masitinib group, except for adverse events leading to death. Subgroup analysis based on specific neurodegenerative diseases consistently demonstrated a higher incidence of adverse events with masitinib treatment in both AD and MS populations. Subgroup analysis based on masitinib dose indicated that the 4.5 mg/kg/d dose was associated with a significantly higher incidence of all grades of adverse events, except for those leading to death. However, the 3 mg/kg/d dose showed no difference between masitinib and placebo regarding severe and non-fatal serious adverse events. This suggests a potential dose-dependent safety profile for masitinib. Notably, the 6 mg/kg/d dose was not included in the subgroup analysis due to heterogeneity in its administration across the included studies.

These safety findings align with RCTs conducted on non-neurological diseases, such as severe asthma, rheumatoid arthritis, and indolent systemic mastocytosis, where adverse events and treatment discontinuation rates were higher in the masitinib group [9–11]. While adverse events were significantly higher in the masitinib groups in our meta-analysis, the relative risk remained relatively modest. Moreover, most adverse events were manageable, considering the promising benefits of masitinib in slowing these fatal neurodegenerative diseases. However, it's essential to recognize that adverse events associated with masitinib can still impact patients' quality of life and may necessitate management or treatment discontinuation. Since the 3 mg/kg/d dose was associated with fewer adverse events, dose adjustment may improve the safety profile of masitinib. However, most RCTs recommended the 4.5 mg/kg/d dose as the optimal dose for maximizing treatment benefits and improving the benefit/risk balance [5, 6, 8]. Additionally, in several trials, the group receiving a fixed dose of 6 mg/kg/day was terminated as recommended by the monitoring committee [5, 6]. Consequently, the 4.5 mg/kg/d dose is the preferred option for upcoming trials. However, further dose-ranging studies are essential to determine the optimal masitinib dosage with minimal safety concerns in patients with neurodegenerative diseases.

While this meta-analysis represents the first comprehensive evaluation of masitinib's safety profile in patients with neurodegenerative diseases, it is important to acknowledge its limitations. The number of included RCTs was relatively small, which may limit the generalizability of our findings. Additionally, variations in disease types and patient populations across the included studies introduce heterogeneity. Future research should address these limitations by conducting larger and more robust RCTs with standardized reporting of adverse events and longer follow-up periods to provide a more comprehensive understanding of masitinib's safety in neurodegenerative diseases.

## Conclusion

The current evidence suggests safety concerns associated with masitinib use in patients with neurodegenerative diseases. While these safety concerns were modest compared to the promising treatment benefits, the adverse events can still impact patients' quality of life and may necessitate management or treatment discontinuation. Further investigations and dose-ranging studies are recommended to determine the optimal effective dose of masitinib with the least safety concerns in patients with neurodegenerative diseases.

### Supplementary Information

Below is the link to the electronic supplementary material.

Supplementary file1 (PDF 306 KB)

## Data Availability

Not applicable.
