# Supplementary material for: Safety of masitinib in patients with neurodegenerative diseases: a meta-analysis of randomized controlled trials
Source: Neurol Sci. 2024 Apr 17;45(7):3503–7. doi: 10.1007/s10072-024-07502-y (PMC11176095; doi:10.1007/s10072-024-07502-y)
Supplement: Supplementary file 1 — Supplementary file1 (PDF 306 KB) [file 10072_2024_7502_MOESM1_ESM.pdf]

# Safety of masitinib in patients with neurodegenerative diseases: a meta-analysis of randomized controlled trials

## *Supplementary file*

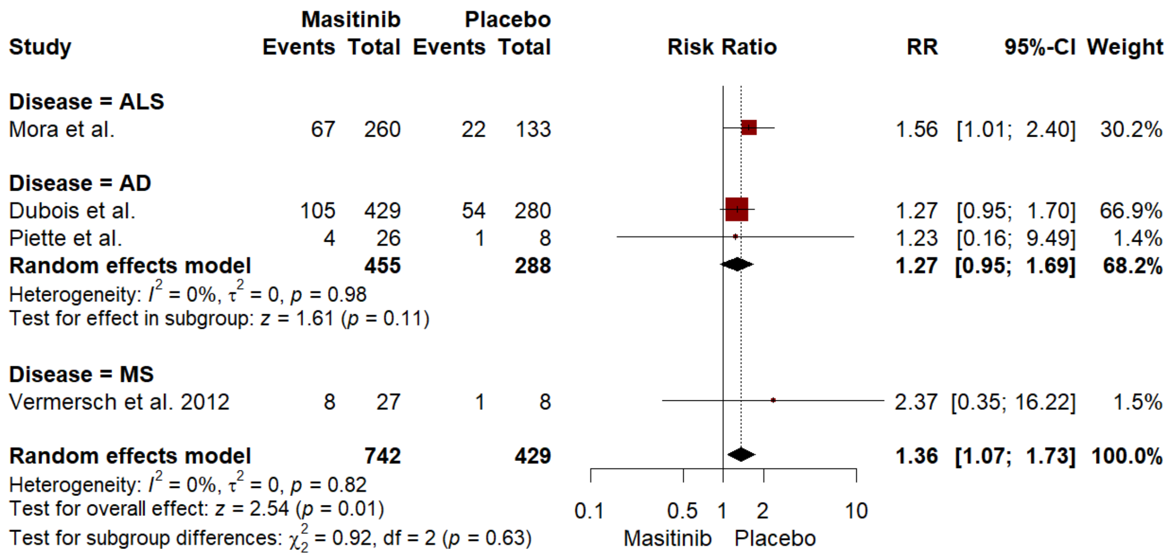


**Supplementary eFigure 1:** Forest plot comparing the overall incidence of severe adverse events, irrespective of masitinib dosage, between the masitinib and placebo groups.

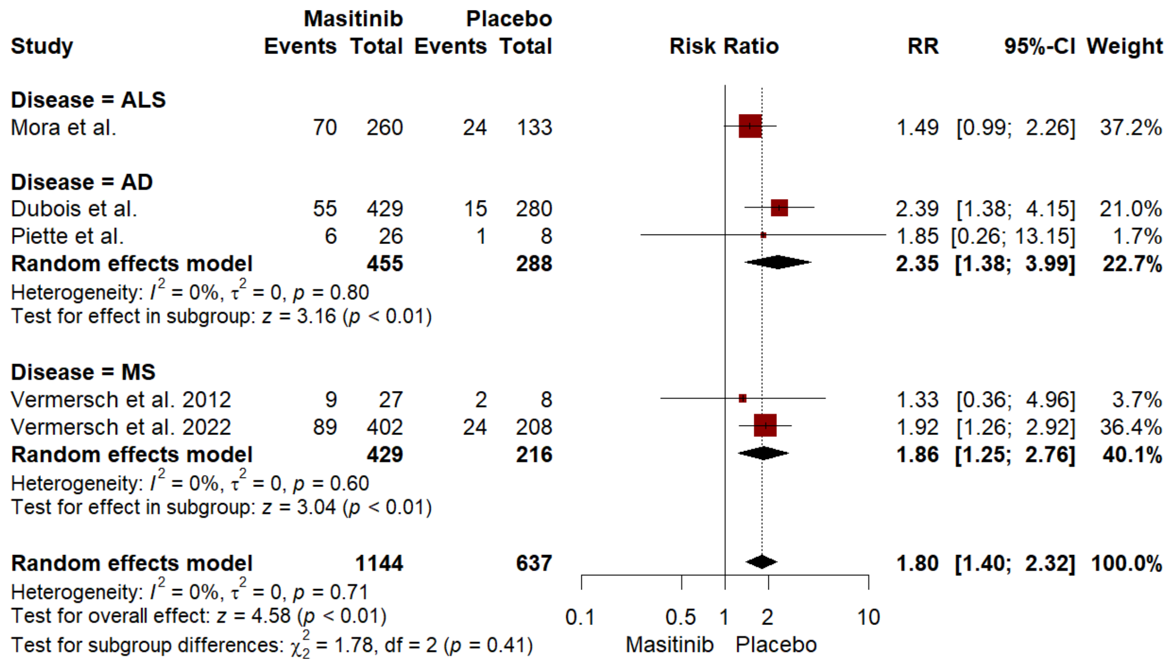


**Supplementary eFigure 2:** Forest plot comparing the overall incidence of non-fatal serious adverse events, irrespective of masitinib dosage, between the masitinib and placebo groups.

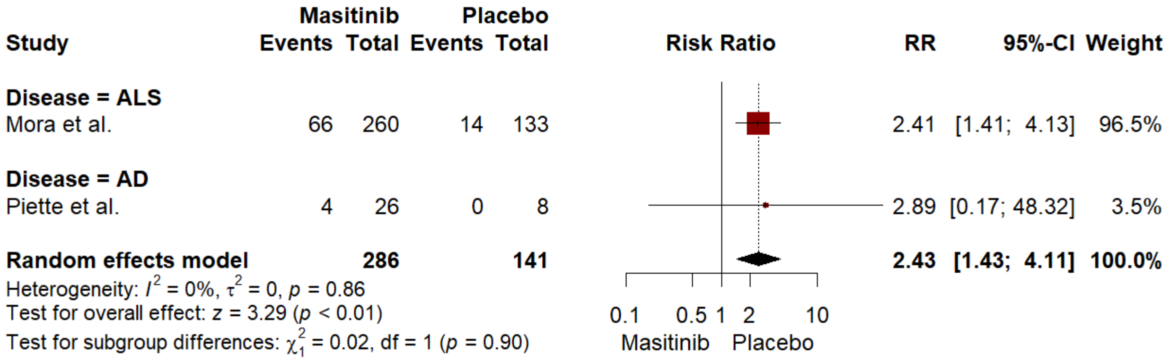


**Supplementary eFigure 3:** Forest plot comparing the overall incidence of adverse events leading to dose reduction, irrespective of masitinib dosage, between the masitinib and placebo groups.

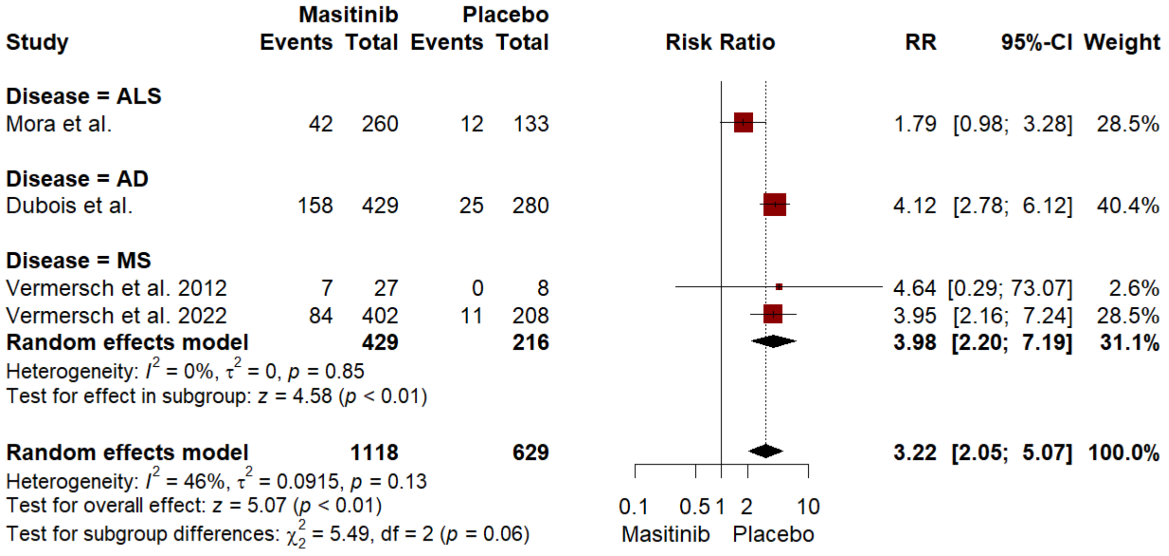


**Supplementary eFigure 4:** Forest plot comparing the overall incidence of adverse events leading to discontinuation, irrespective of masitinib dosage, between the masitinib and placebo groups.

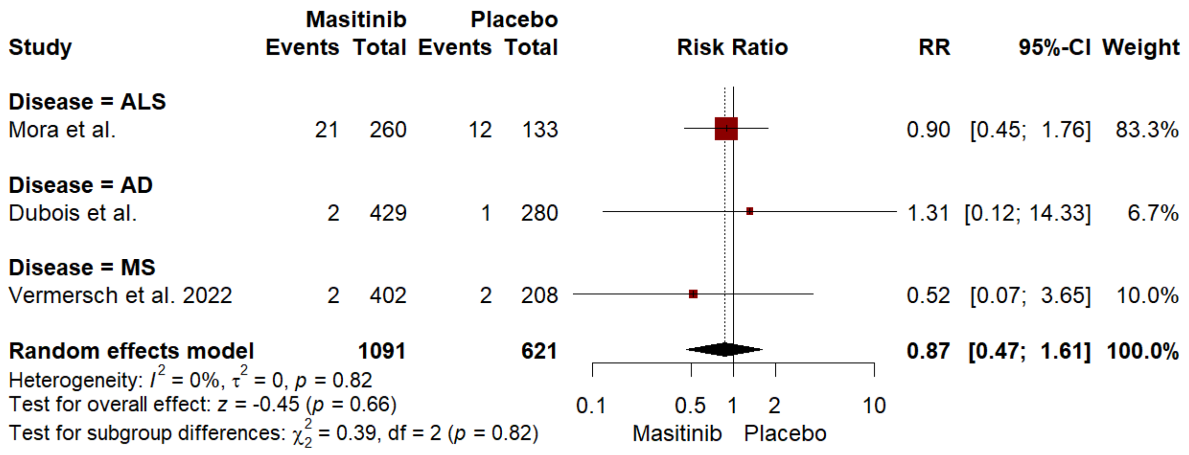


**Supplementary eFigure 5:** Forest plot comparing the overall incidence of adverse events leading to death, irrespective of masitinib dosage, between the masitinib and placebo groups.

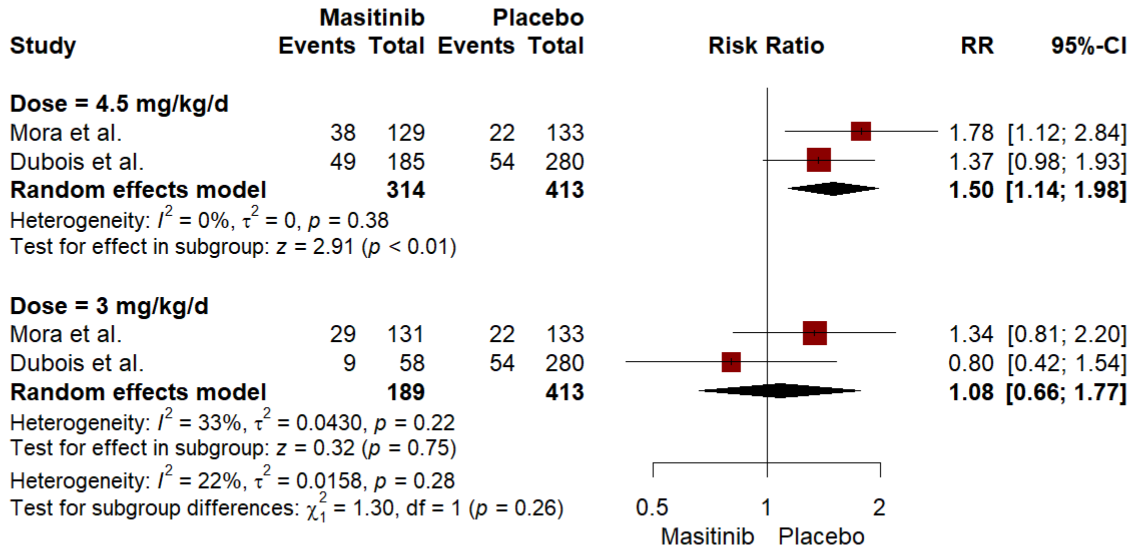


**Supplementary eFigure 6:** Forest plot of subgroup analysis for masitinib dosage, comparing the combined incidence of severe adverse events between the masitinib and placebo groups.

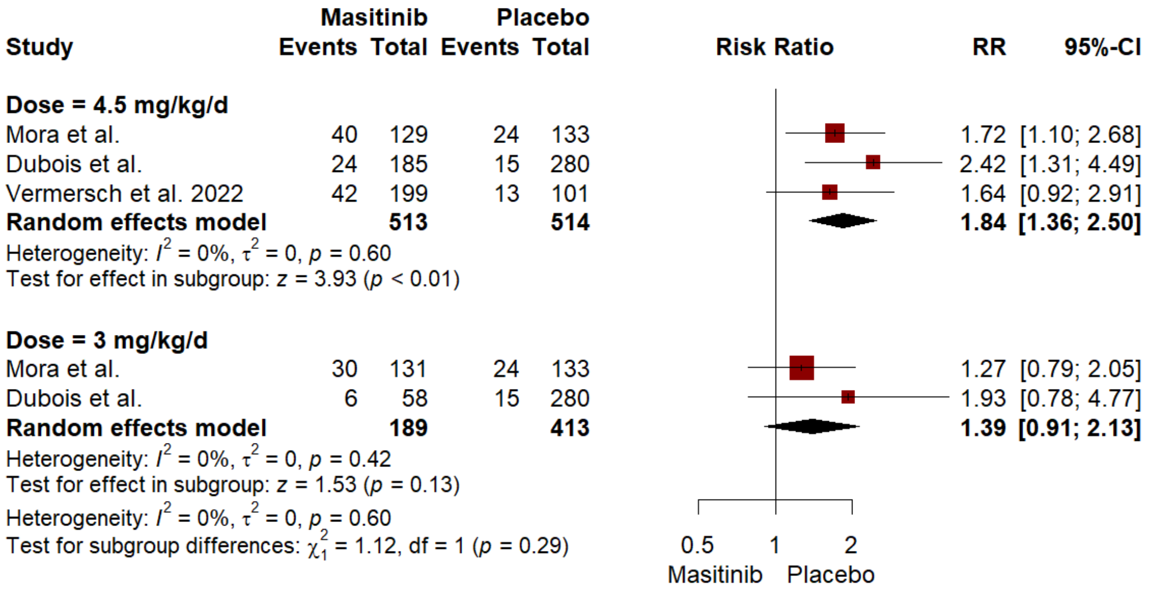


**Supplementary eFigure 7:** Forest plot of subgroup analysis for masitinib dosage, comparing the combined incidence of non-fatal serious adverse events between the masitinib and placebo groups.

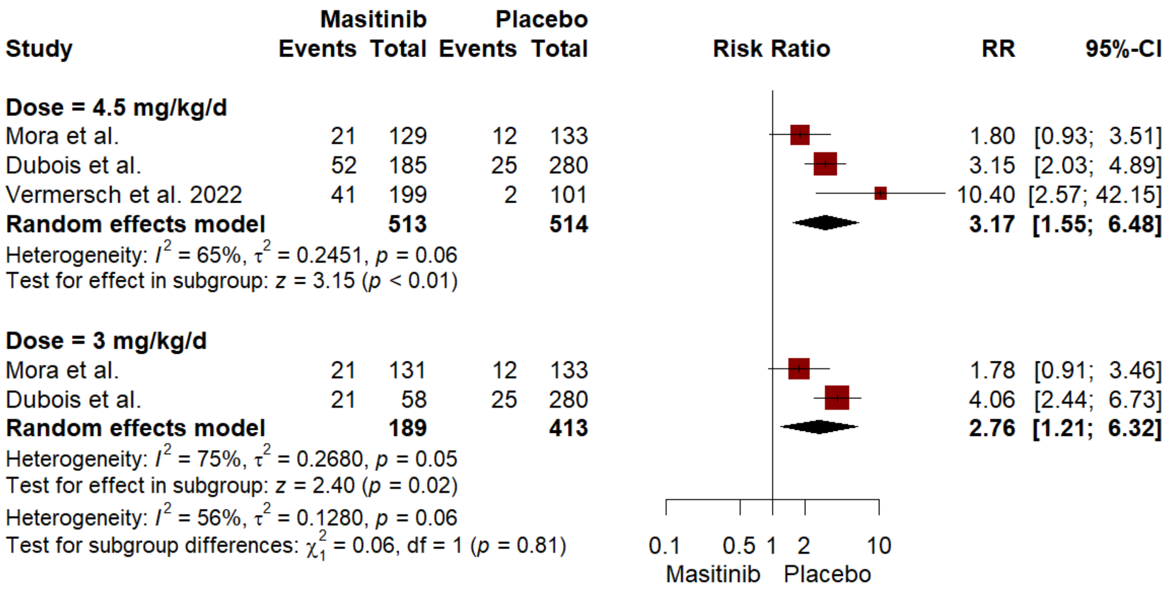


**Supplementary eFigure 8:** Forest plot of subgroup analysis for masitinib dosage, comparing the combined incidence of adverse events leading to discontinuation between the masitinib and placebo groups.

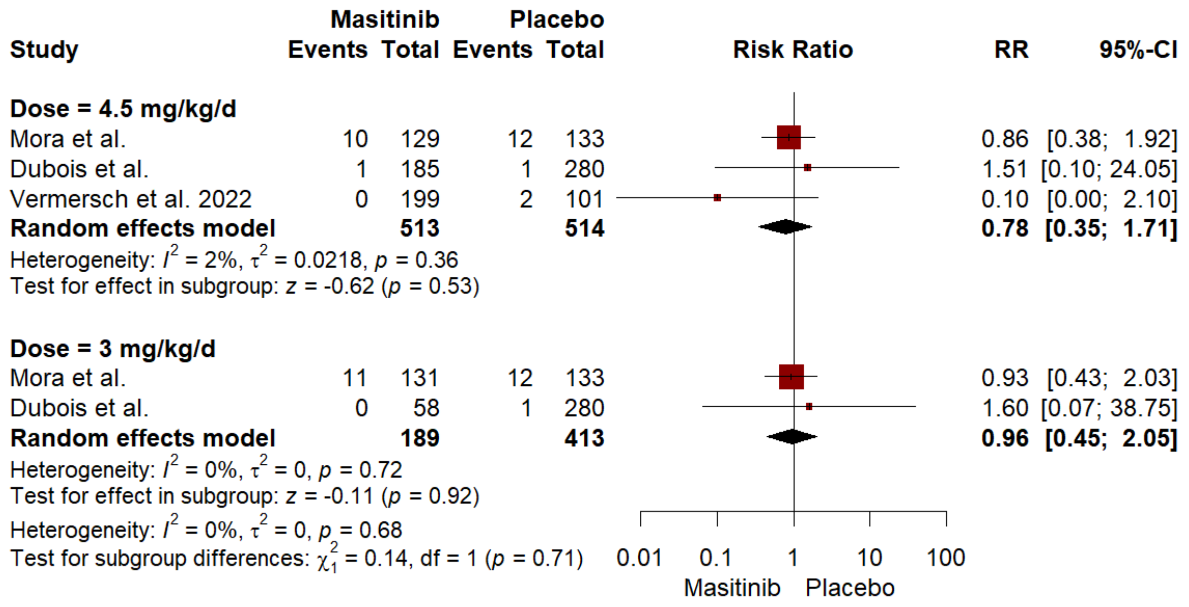


**Supplementary eFigure 9:** Forest plot of subgroup analysis for masitinib dosage, comparing the combined incidence of adverse events leading to death between the masitinib and placebo groups.
